# Iliocaval and iliofemoral venous stenting for obstruction secondary to tumor compression

**DOI:** 10.1186/s42155-024-00438-6

**Published:** 2024-03-22

**Authors:** Ahmed K. Aly, Amgad M. Moussa, Olivier Chevallier, Sirish Kishore, Elena Petre, Adie Friedman, Yolanda Bryce, Adrian Gonzalez, Juan Camacho, Ernesto Santos, Fourat Ridouani

**Affiliations:** 1 Radiology, Division of Interventional Radiology, MedStar Health, Baltimore, USA; 2 Radiology, Division of Interventional Radiology, Memorial Sloan Kettering Cancer Center, New York, USA; 3 Department of Vascular and Interventional Radiology, Image-Guided Therapy Center, François-Mitterrand University Hospital, Dijon, France; 4 Radiology, Division of Interventional Radiology, Stanford Healthcare, Stanford, USA; 5 Radiology, Division of Interventional Radiology, Sarasota Memorial Health Care System, Sarasota, USA

**Keywords:** Venous stenting, Deep venous obstruction, Mechanical thrombectomy, Cancer, Venous compression

## Abstract

**Background:**

Cancer patients with pelviabdominal masses can suffer from lower extremity symptoms due to venous compression. The effectiveness of venous stenting has been established in extrinsic venous compression in benign conditions like May-Thurner syndrome. In this retrospective study we evaluate the efficacy and safety of caval, iliocaval and iliofemoral venous stenting for cases of extrinsic venous compression caused by malignant masses in cancer patients.

**Methods:**

IRB-approved retrospective review of patients who underwent iliofemoral venography with venoplasty and stenting between January 2018 and February 2022 was performed. Patients with extrinsic venous compression caused by malignant masses were included. Data on patient demographics, pre-procedure symptoms, procedural technique, stent characteristics, outcomes and follow-up were collected. Descriptive statistics were used to assess technical success, clinical success, primary stent patency and adverse events of the procedure.

**Results:**

Thirty-seven patients (19 males, 18 females) who underwent 45 procedures were included. Deep venous thrombosis (DVT) was present in 21 (57%) patients. Twenty-nine patients (78%, 95% CI 62–90%) reported clinical improvement of the presenting symptoms. The median overall survival after the procedure was 4.7 months (95% CI 3.58–5.99). Eight (22%) patients were alive at last follow up with median follow up of 10.33 months (Range 2–25 months). Twenty-six patients had patent stents on their last follow up imaging (70%, 95% CI 61%-91%). Two patients had a small access site hematoma which resolved spontaneously. Two patients developed moderate, and 1 patient developed severe adverse events related to post procedure therapeutic anticoagulation.

**Conclusion:**

Venous stenting is a safe procedure and should be considered as part of the palliative care for patients with debilitating lower extremity symptoms related to iliocaval and iliofemoral venous compression.

**Graphical Abstract:**

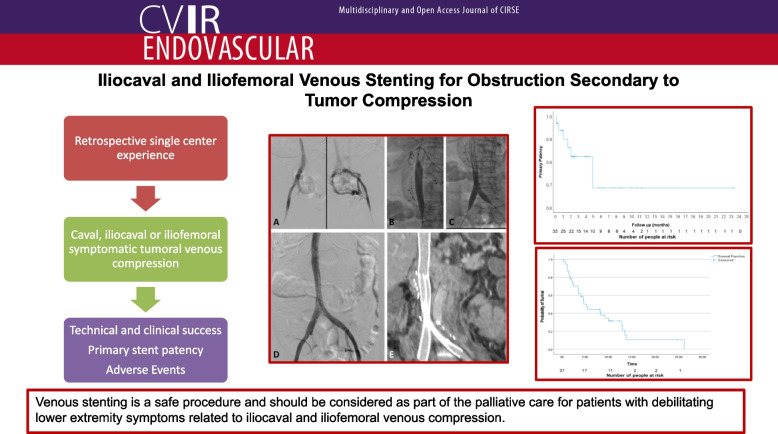

## Background

Neoplastic pelvic and abdominal masses can compromise the lower extremity venous drainage in multiple ways. The mere presence of malignancy increases the risk of venous thrombosis due to associated hypercoagulable state [1, 2]. Extrinsic vessel compression or invasion by pelviabdominal masses is another important contributor, which sometimes is associated with deep venous thrombosis (DVT) [3, 4]. Inferior Vena Cava (IVC) or iliofemoral venous compression in those situations can present with symptoms of lower limb venous congestion including swelling, pain, ulcer, skin hyperpigmentation, varicosities, and venous claudication, which can greatly affect quality of life [5, 6] Other factors contributing to the lower limb symptoms in this patient population include lymphedema following surgery or radiotherapy, liver compromise and heart failure [7]. Metastatic lymphadenopathy associated with some pelviabdominal masses can also contribute to lower extremity lymphedema [8, 9].

Endovascular venous reconstruction has proven efficacy in decreasing patients’ lower limb venous congestion symptoms through restoring outflow [10–16]. Since the advent of dedicated venous stents and thrombectomy devices data about venous stenting in this population of venous compression by neoplastic masses is limited to case reports and series [17].

In this retrospective study we evaluated the safety, technical success and clinical outcomes of IVC, Iliocaval and/or iliofemoral venous stenting in the treatment of venous obstruction caused by abdominal and pelvic tumor compression in addition to identifying anatomic and procedural factors influencing clinical success.

## Methods

### Study population

This retrospective single center study was approved by the institutional review board. All venous interventions were reviewed from January 2018 to February 2022. Patients with caval, iliocaval or iliofemoral narrowing of more than 70% on cross sectional imaging or occlusion due to extrinsic compression or invasion by adjacent abdominal/pelvic masses in addition to venous compression related symptoms (swelling, pain, redness) were included. Dedicated CT venogram of the abdomen and pelvis was obtained in all patients to delineate the extent of the stenosis. Ultrasound examination of the lower extremity veins was used to evaluate for deep venous thrombosis in the lower extremities. Exclusion criteria included patients with May-Thurner pathology and patients with venous obstruction without an underlying malignant mass. Patients with no imaging follow up were excluded from stent patency analysis.

### Outcomes

Medical records were reviewed to collect patients’ demographic characteristics, cancer diagnosis, presenting symptoms, presence of DVT, pre-procedure anticoagulation, history of radiotherapy to the obstructed region, level of obstruction, procedure technical details, post procedure outcomes, duration of follow up, stent patency and patient survival.

Post procedure clinical outcomes were collected by reviewing the follow up notes from interventional radiology and other clinical services. Due to heterogeneity of follow up scores used, change in symptoms (including pain, lower extremity swelling, pruritis, skin changes) was described and classified into 1- Worsening of presenting symptoms, 2- No change in symptoms, 3- Mild subjective improvement, 4- Marked improvement of the presenting symptoms. Technical Success was defined as the ability to cross the area of venous obstruction and successfully place venous stent with restoration of in-line flow, restoration of near native vein diameter and disappearance of collaterals at the end of the procedure. Clinical success was defined as Mild or Marked improvement of patient’s presenting symptoms.

Post procedure primary patency was evaluated by reviewing follow up imaging and the dates and types of the last study showing patent stents and first study showing occluded stents were collected when available. Post procedure adverse events were described and classified according to the SIR adverse event classification [18].

### Procedure

All procedures were performed by 8 interventional radiologists with 2–15 years of experience. Venous access site was determined based on the distal extension of the obstruction/narrowing and the thrombus (if present), ultrasound guided access was obtained using micro puncture access kit (Cook, Bloomington, IN, USA) into the femoral, popliteal or below knee veins either unilaterally or bilaterally depending on the location of the obstruction. In cases where the obstruction was not amenable to crossing from the distal access site or there was a concern about migration of IVC stent during deployment an additional right Jugular access was obtained for through and through access.

**Fig. 1:**
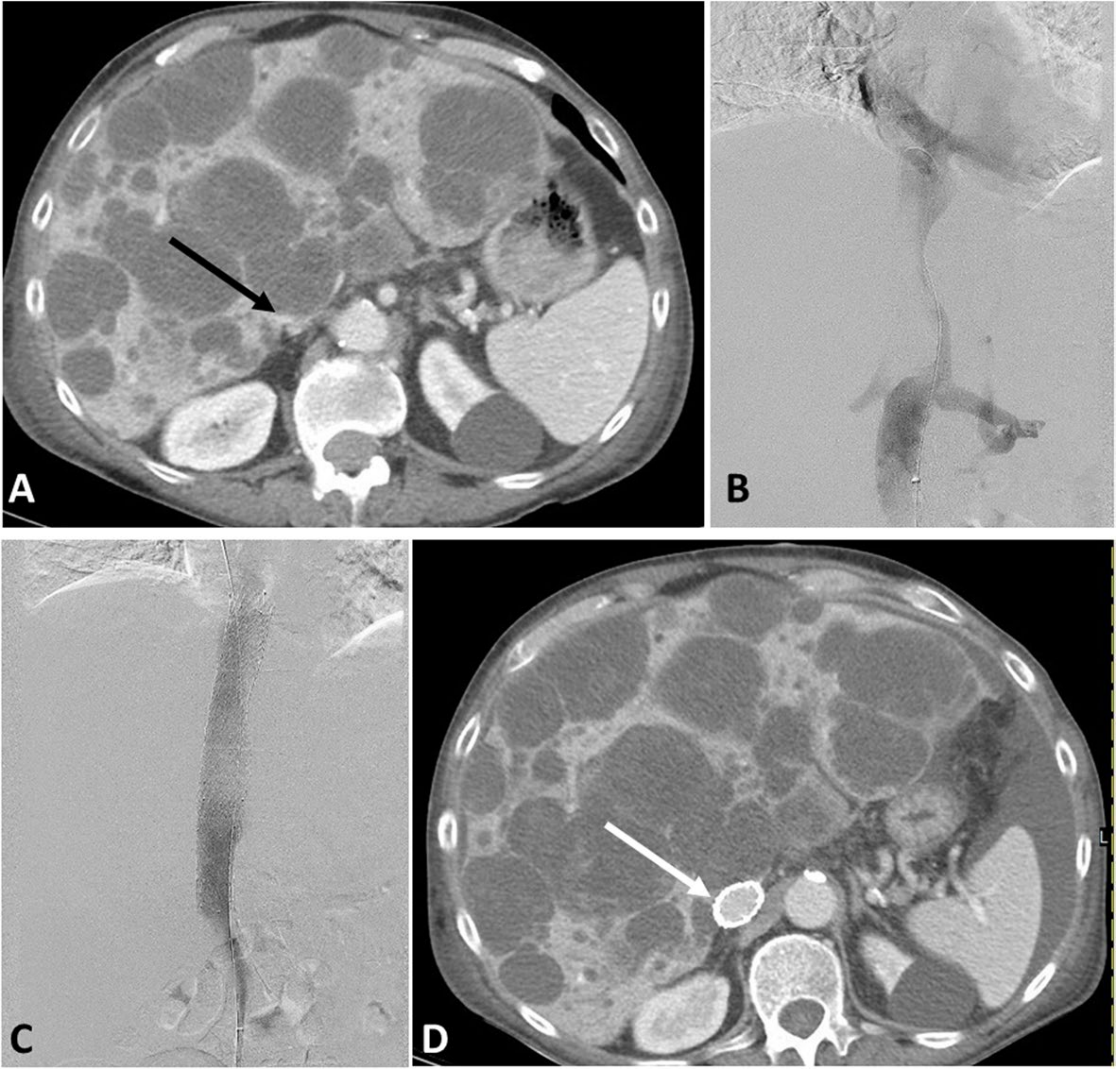
79 y male with metastatic chondrosarcoma presented with new onset of bilateral lower extremity swelling. **A** CT of the abdomen showing growing hepatic metastasis compressing the intrahepatic IVC (Black arrow). **B** Venography showing attenuation of the intrahepatic IVC. **C** Post stenting venography showing successful restoration of flow with disappearance of collaterals after placing 20 mm stent and post-stenting venoplasty using 20 mm balloon. **D** CT of the abdomen obtained one week later due to persistent abdominal pain showing patent stent and new bleeding into some of the hepatic lesions (not shown). Lower extremity swelling already improved

**Fig. 2:**
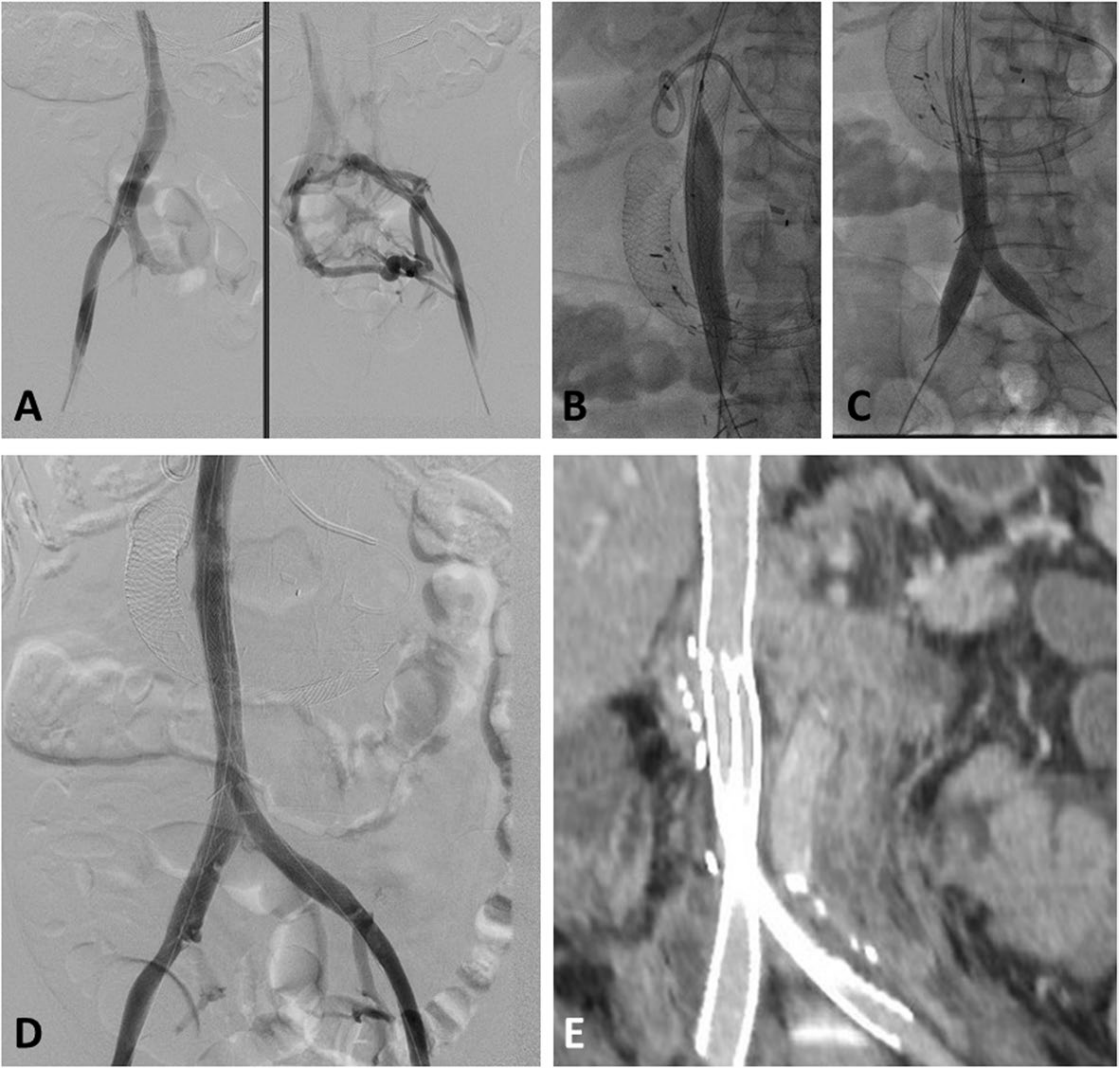
60 Y Female with RCC presenting with new lower extremity swelling due to Iliocaval compression by retroperitoneal masses **A** Initial venogram images obtained by bilateral femoral vein injection showing iliocaval obstruction with multiple collaterals **B** 24 mm Wall Stent deployed in the IVC with post stenting dilatation with 22 mm balloon. **C** bilateral kissing 18mm Iliocaval Wall stents deployed followed by stent dilatation with 16 mm balloon. **D** Final venogram showing restoration of in-line flow. E) Curved reconstruction of coronal follow up CT image showing preserved stent patency

Venography was performed to delineate the anatomy of the obstruction (Figs. 1 and 2). The obstruction was crossed using a combination of 4 Fr/5 Fr catheter and 0.035 crossing wire. When DVT was present pharmaco-mechanical or pure mechanical thrombectomy was performed according to the operator’s preference. In Acute DVT cases where the thrombus was not cleared by thrombectomy, an infusion catheter was placed, and overnight thrombolysis was performed. Intravascular ultrasound (IVUS) was used in some cases to better delineate the extension and degree of the obstruction. IVC, Iliocaval and/or iliofemoral stent placement was then performed. Multiple stent types were used depending on operator preference including Wall stent (Boston Scientific, Marlborough, MA, USA), Venovo (Bard/Becton, Dickinson and Company, Tempe, Arizona, USA), Vici (Boston Scientific, Marlborough, MA, USA), SMART (Cordis Corp, Fremont, CA, USA), Zilvervena (Cook, Bloomington, IN, USA) and/or Viabahn stent graft (WL Gore and Associates, Flagstaff, AZ, USA). Post-stenting balloon dilatation was performed when necessary to dilate the stents to target diameter.

### Statistical analysis

Clinical characteristics of the study cohort and procedure details were summarized with descriptive statistics. The proportions of technical success, clinical success and patent stents on last follow up were calculated with 95% confidence intervals. The effect of different factors on clinical success was evaluated using Chi squared test or Fisher exact test as appropriate. Patients’ survival and stent patency was described by the Kaplan–Meier method. IBM SPSS Statistics for Windows, version 26 (IBM Corp., Armonk, N.Y., USA) was used for statistical analysis. Adverse events after the procedure were reported according to the Society of Interventional Radiology classification [18].

## Results

Between January 2018 to February 2022, 45 procedures were performed for 37 patients with caval, iliac or iliofemoral venous obstruction related to malignant pelviabdominal masses. Prostate and Gynecologic malignancies were the most common diagnoses. New or increased lower extremity swelling was the most common presenting symptom. Twenty-one patients (56.7%) had DVT at the time of presentation and were started on preprocedural anticoagulation. 14 patients (37.8%) had history of radiotherapy to the site of venous compression. Baseline characteristics are summarized in Table 1.

**Table 1:** Baseline patient characteristics

Age (y)	63 (56–75)
Gender	
• Male	19 (51)
• Female	18 (49)
Cancer Diagnosis	
• Prostate	8 (21.6)
• Gynecologic	9 (24.3)
• Colorectal	3 (8.1)
• Renal	4 (10.8)
• Urinary Bladder	3 (8.1)
• Other^a^	10 (27.1)
Level of compression	
• Inferior Vena Cava	6 (16.2)
• Iliocaval	8 (21.6)
• Iliac	20 (54.1)
• Iliofemoral	3 (8.1)
Presenting Symptoms	
• Unilateral Swelling	23 (62%)
• Bilateral Swelling	14 (38%)
• Lower extremity Pain	11 (30%)
• Other^b^	4 (11%)
Presence of DVT	
• None	16 (43.2)
• Bland thrombus	19 (51.4)
• Tumor thrombus	2 (5.4)
Pre-procedure AC	
• None	16 (43.2)
• Enoxaparin	12 (32.4)
• Heparin drip	2 (5.4)
• Other^c^	7 (19)

Statistics shown are median (IQR: interquartile range) or n(%)

^a^Other cancers included Breast, Chondrosarcoma, Hepatocellular, Lymphoma, Melanoma, Mesothelioma, Multiple Myeloma, Penile SCC, Sarcoma, PEComa

^b^Other symptoms included itching, redness, cramping, venous ulcers, varicosities

^c^Other anticoagulants included Fondaparinux (2), Apixaban (3), Rivaroxaban (2)

### Procedure details

Thrombolytic injection was performed in 5 cases, 4 of which were as a part of intra-procedure pharmaco-mechanical thrombectomy using Angio Jet (Boston Scientific, Natick, MA, USA)). One patient required overnight thrombolytic infusion. Mechanical thrombectomy was used in 11 procedures, 2 of which required more than one device. Balloon dilatation was mostly used after stent placement. Primary venoplasty was performed in 17 (37%) procedures and was always followed by venous stenting. A combination of mechanical thrombectomy followed by primary venoplasty was performed in 4 procedures (3 patients). IVUS was used in 11 procedures (25%) and was based on operator’s preference and the reliability of final venogram to evaluate adequate stent placement.

Five patients (6 procedures) had isolated IVC compression. Thirteen patients required bilateral stenting on their initial procedure and 19 patients required unilateral stenting. For Iliocaval and iliofemoral venous reconstruction, a combination of more than one stent type was used in 3 procedures on the right side and 4 procedures on the left. The choice of stent diameter varied depending on the venous segment involved. Procedure details are summarized in Table 2.

**Table 2:** Procedure technical details

Access			
• Popliteal	18		
• Femoral	21		
• Saphenous	3		
• Jugular for additional access	10		
• Jugular only	3		
Thrombectomy	11 procedures		
• AngioJet	6		
• INARI	2 ClotTriever, 1 FlowTreiver		
• CLEANER	2		
• Other	2		
Stent type	**IVC**	**Iliac**	**Iliofemoral**
• WallStent	12	12	5
• Venovo	1	2	0
• Vici	0	11	5
• S.M.A.R.T	0	5	3
• Other	0	1	2
Stent Diameter by level			
• IVC	24 mm (22–24)		
• Iliocaval	16 mm (14–16)		
• Iliac	14 mm (12–16)		
• Iliofemoral	12 mm (12–14)		
IVUS use	11 (24.4)		

Statistics shown are median (IQR: interquartile range) or n(%)

*IVC* Inferior Vena Cava, *IVUS* Intravascular ultrasound

### Clinical outcomes and follow up

Technical success was 100% with restoration of flow and disappearance of collaterals at the end of the procedure in all patients (Figs. 1 and 2). Clinical success was 78% (95% CI 62–90%) with mild improvement reported in 13 and marked improvement reported in 16 patients. Five patients had unchanged, and 3 patients had worsening symptoms. Post procedure anticoagulation was prescribed for 35 patients. Enoxaparin was the most used in 23 patients, followed by direct oral anticoagulant in 8 patients.

**Fig. 3:**
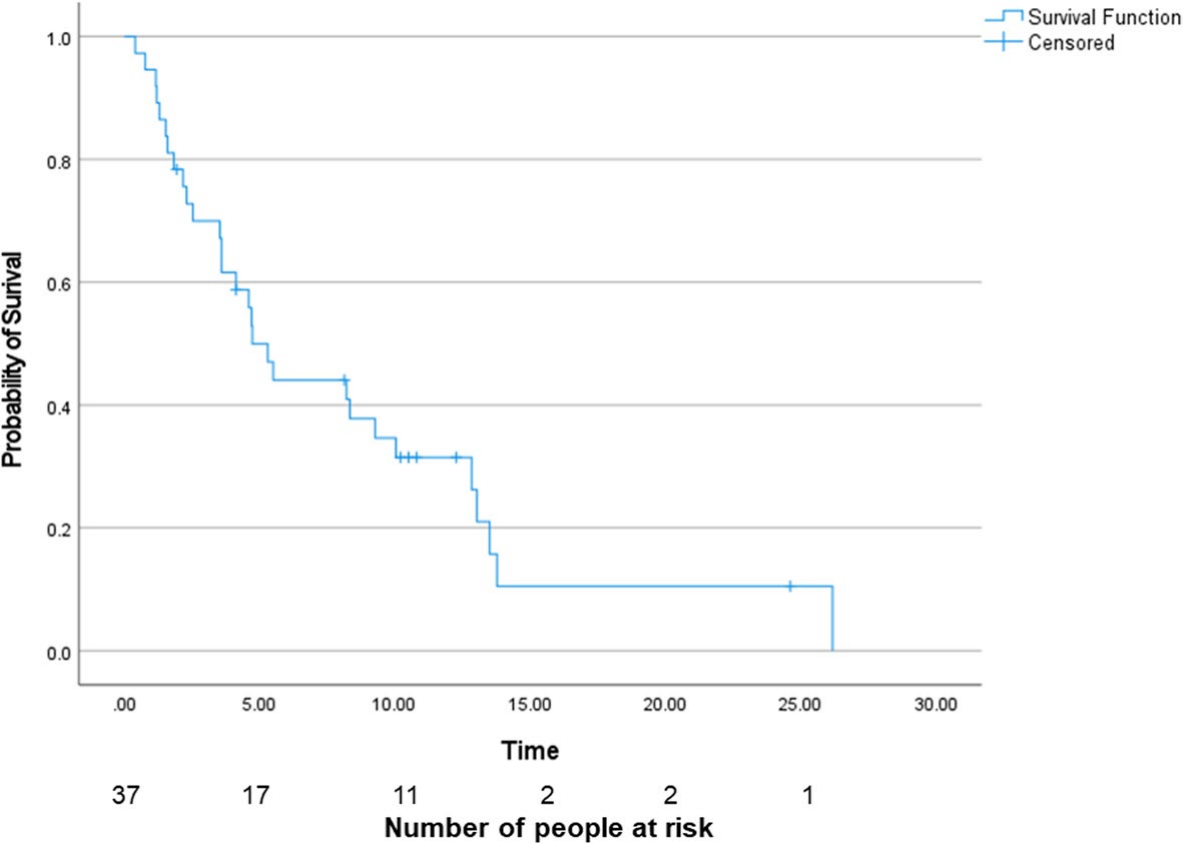
Kaplan–Meier curve of patient’s survival after endovascular venous stenting in months

**Fig. 4:**
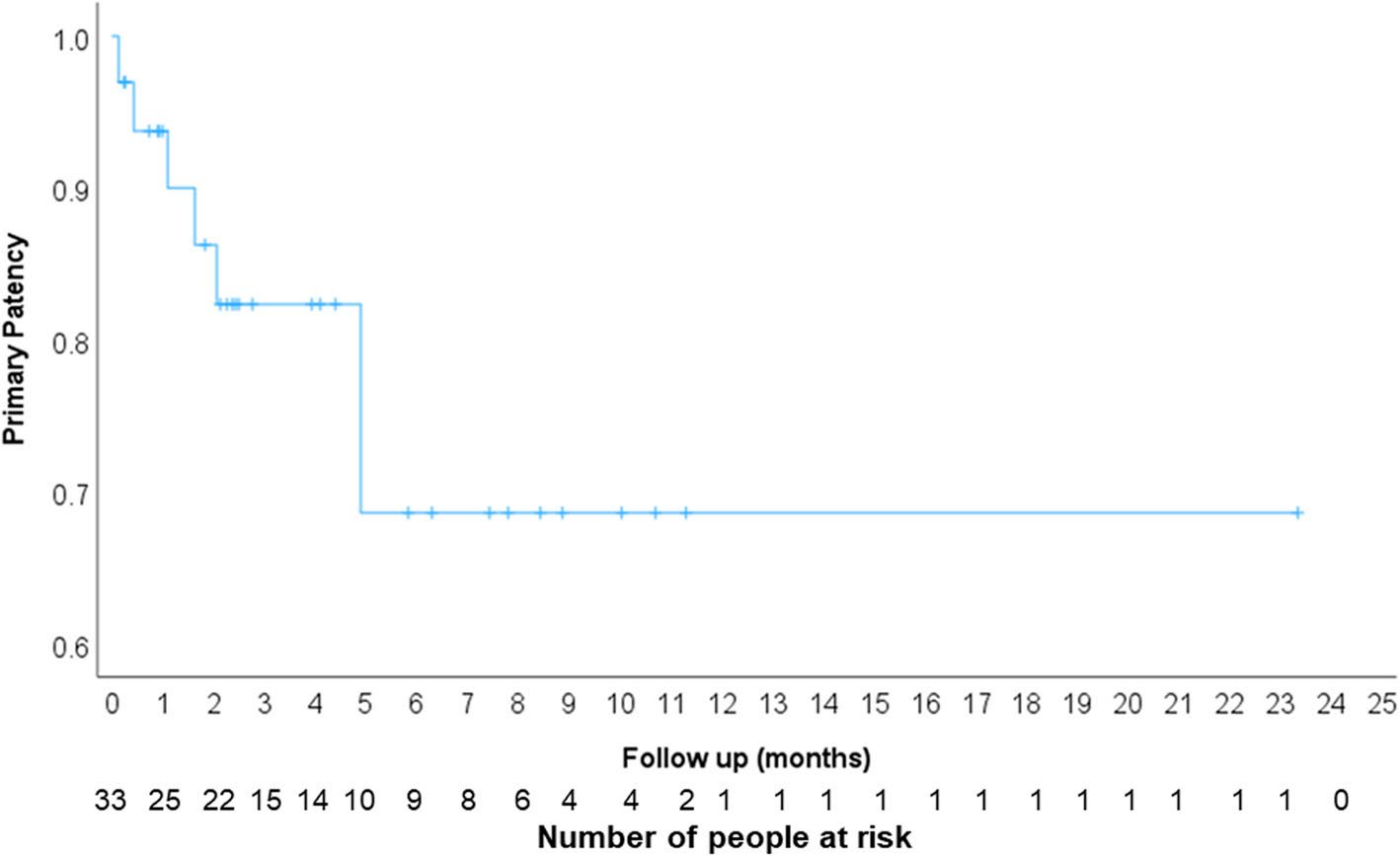
Kaplan–Meier curve of the placed stents’ primary patency in months

Twenty-four patients were deceased by 1 year (77.4%) with median overall survival of 4.70 months (95% CI 3.58–5.99) (Fig. 3). Four patients did not have any follow up imaging in the chart to evaluate stent patency and 26 patients had patent stents on their last follow up imaging (79%, 95% CI 61%-91%). Primary patency of the placed stents at 1, 3 and 6 months was 93%, 81% and 69% respectively (Fig. 4). In the 7 patients who had their stents occluded, median time to occlusion was 49 days (Range 4–68 days). In this group, 4 patients had stent occlusion due to external tumor compression, 2 patients developed in-stent thrombosis and 1 patient had both. The clinical success rate and patency on last follow up imaging was not affected by treated venous segment, used stent type, presence of baseline thrombus or history of radiotherapy (Table 3). The median follow-up duration for the study population was 4.13 months (Range 0.73–24.60 months). Most common imaging study available to evaluate stents was contrast enhanced CT (Table 3).

**Table 3:** Post procedure outcomes

Technical Success	100% (95% CI 90.5–100%)
**Clinical Success**	29/37(78%) (95% CI 62–90%)
Venous segment	*P* = 0.22
• IVC	4/5 (80%)
• Iliac/Iliocaval	18/21 (86%)
• Iliofemoral	7/11 (64%)
Laterality	*P* = 0.06
• Unilateral	13/20 (65%)
• Bilateral	12/12 (100%)
Stent type	*P* = 0.4
• Nitinol stent	9/13 (69%)
• Wall Stent	13/16 (81%)
• Combination	6/7 (86%)
• Covered stent	1/1 (100%)
Dedicated venous stent^a^	*P* = 0.7
• Yes	7/9
• No	15/20
• Combination	7/8
DVT	*P* = 0.6
• Tumor thrombus	1/2(50%)
• Bland thrombus	16/20(80%)
• No thrombus	12/15(80%)
RTH	*P* = 0.4
• Yes	10/14(71%)
• No	19/23(82.6%)
Survival	
• Median Survival	4.7 months (95% CI 3.58–5.99)
• 30-day mortality	5.4%
• 1 year mortality	77.4%
**Proportion of patent stents**	26 (79%, 95% CI 61%-91%)
Venous segment	*P* = 0.28
• IVC	2/4
• Iliac/Iliocaval	16/20
• Iliofemoral	8/9
Stent type	*P* = 0.88
• Nitinol stent	9/11
• Wall Stent	11/15
• Combination	5/6
• Covered stent	1/1
DVT	*P* = 0.57
• Tumor thrombus	1/2(50%)
• Bland thrombus	14/17(82%)
• No thrombus	11/14(79%)
RTH	*P* = 1.0
• Yes	11/14(79%)
• No	15/19(79%)
Type of last follow up imaging	
• US	7
• CT with contrast	22
• CTV	10
• MRI with contrast	1

*IVC* Inferior Vena Cava, *DVT* Deep Venous Thrombosis, *RTH* Radiotherapy, *CTV* CT venography

^a^Dedicated venous stents included the Vici and Venovo stents

### Adverse events

Two patients developed small access site hematoma which resolved spontaneously. No procedure related major adverse events occurred. Five bleeding events were reported during follow up, which are likely due to anticoagulation. Two patients had bleeding from their known hepatic metastasis at 1- and 7-days post procedure, both required anticoagulation to be temporarily stopped. The first patient stabilized after transfusing 1 unit of packed red blood cells. The second patient did not suffer a significant hemoglobin drop and was managed conservatively. One patient had spontaneous epidural hematoma in the lower spine that required surgical evacuation and laminectomy 4 days after the procedure. Two patients developed spontaneous muscular hematoma in the lower extremities few months after the procedure which did not need intervention.

Two patients were deceased by 30 days (5.4%) from the first intervention. Both died of hypoxic respiratory failure; the first due to culture positive pneumocystis pneumonia, and the second developed repeated pleural effusion related to progression of disease.

## Discussion

In this study, venous stenting performed for patients with lower extremity symptoms associated with malignant venous compression in the caval, iliocaval or iliofemoral venous segments showed a high clinical success rate of 78% with high primary patency at last follow up.

The survival of patients in the study population was rather short (Median 4.7 months, Mean 6.9 months (Range 0.4–26.2 months)), which was likely due to their poor prognosis given the advanced stage of their malignancy. This is in line with the reported survival rates in the literature. O’Sullivan et al. reported a mean survival of 7.5 months (Range 0.15–36 months)) [7] and Maleux et al. reported median survival close to 6 months with 30-day and 1-year mortality of 15.79% and 80.2% respectively [5]. Similar results were also reported by Perez-Johnston et al. as they reported 50% mortality during their follow up with mean survival of 6.8 months [19].

Due to the short survival of those patients, the focus of venous intervention in this situation is more towards improving the quality of life. Restoration of flow is almost always feasible during the procedure with reported technical success rates of 100% [5, 7, 20]. Clinical success, however, is not always achieved due to the other contributing factors. Our reported clinical success rate of 78% is similar to that reported in the literature, which is between 80–100% [5]. Even in situations with short life expectancy, select cases can benefit from the procedure. In this study, patients who died within 30 days of the procedure reported improvement of their symptoms which again points toward the palliative value of venous stenting.

The patency of Iliocaval and iliofemoral venous stents can usually be maintained for over 2 years [21, 22]. Due to the short survival of this patient population, a high percentage of stents were expected to remain patent till the patient’s death. Seventy-nine percent of the patients in our study were found to have patent stents on the last available follow up imaging with no additional procedures. Other studies reported patency rates of 63–83% [5, 7, 23]. Most common mode of stent failure in our study was recurrent external compression by the tumor (57%), this is similar to what Perez-johnston etal reported in their cohort where 50% of the stent occlusion was related to tumor external compression [19]. Drabkin et al. showed that anticoagulation was associated with longer duration of primary patency. In our study, all patients were started or continued on therapeutic anticoagulation after the procedure [23].

The venous stenting procedure itself was found to be safe, as there were no complications related directly to stent placement, such as stent fracture or migration which were reported in older studies [24]. The more serious complications that were encountered were mostly related to therapeutic anticoagulation use. Whether anticoagulation related complications should be considered a direct effect of stent placement is not easy to determine as some of those patients are to be started on anticoagulation regardless of stenting due to DVT development.

The limitations of the study are mainly due to its retrospective nature, which limits consistent objective evaluation of patients’ symptoms and the use of standardized scoring systems. Lack of use of standardized scoring system increases the subjectivity of clinical success evaluation. The long-term change in symptoms could not also be reliably evaluated. Another limitation is related to the small number of patients that didn’t allow the use of time to event analysis to compare duration of stent patency among different subgroups.

In conclusion, venous stenting for lower limb venous congestion related to abdominopelvic tumor obstruction is a safe procedure that can provide patients with symptomatic relief.

## Abbreviations

DVT: Deep venous thrombosis
IVUS: Intravascular ultrasound
RTH: Radiotherapy
IVC: Inferior Vena Cava
CTV: CT venography
